# Exploring the potential of a multi-level approach to improve capability for continuous organizational improvement and learning in a Swedish healthcare region

**DOI:** 10.1186/s12913-018-3129-3

**Published:** 2018-05-24

**Authors:** M. E. Nyström, E. Höög, R. Garvare, M. Andersson Bäck, D. D. Terris, J. Hansson

**Affiliations:** 1 Department of Learning, Informatics, Management and Ethics, Medical Management Centre, Karolinska Institutet, SE 171 77 Stockholm, Sweden; 2 Department of Public Health and Clinical Medicine, Epidemiology and Global Health, Umeå University, SE 901 87 Umeå, Sweden; 3 Department of Business Administration, Technology and Social Sciences, Luleå University of Technology, SE 971 87 Luleå, Sweden; 4 Department of Social work, Gothenburg University, Box 100, SE 405 30 Gothenburg, Sweden; 5 Center for Family Research, University of Georgia, 1095 College Station Rd, Athens, GA 30602 USA; 6 Department of Public Health Analysis and Data Management, Public Health Agency of Sweden, SE 171 82 Solna, Sweden

**Keywords:** Continuous quality improvement, Organizational learning, Change management, Organizational development, Health care, Social services

## Abstract

**Background:**

Eldercare and care of people with functional impairments is organized by the municipalities in Sweden. Improving care in these areas is complex, with multiple stakeholders and organizations. Appropriate strategies to develop capability for continuing organizational improvement and learning (COIL) are needed. The purpose of our study was to develop and pilot-test a flexible, multilevel approach for COIL capability building and to identify what it takes to achieve changes in key actors’ approaches to COIL. The approach, named “Sustainable Improvement and Development through Strategic and Systematic Approaches” (SIDSSA), was applied through an action-research and action-learning intervention.

**Methods:**

The SIDSSA approach was tested in a regional research and development (R&D) unit, and in two municipalities handling care of the elderly and people with functional impairments. Our approach included a multilevel strategy, development loops of five flexible phases, and an action-learning loop. The approach was designed to support systems understanding, strategic focus, methodological practices, and change process knowledge - all of which required double-loop learning. Multiple qualitative methods, i.e., repeated interviews, process diaries, and documents, provided data for conventional content analyses.

**Results:**

The new approach was successfully tested on all cases and adopted and sustained by the R&D unit. Participants reported new insights and skills. The development loop facilitated a sense of coherence and control during uncertainty, improved planning and problem analysis, enhanced mapping of context and conditions, and supported problem-solving at both the individual and unit levels. The systems-level view and structured approach helped participants to explain, motivate, and implement change initiatives, especially after working more systematically with mapping, analyses, and goal setting.

**Conclusions:**

An easily understood and generalizable model internalized by key organizational actors is an important step before more complex development models can be implemented. SIDSSA facilitated individual and group learning through action-learning and supported systems-level views and structured approaches across multiple organizational levels. Active involvement of diverse organizational functions and levels in the learning process was facilitated. However, the time frame was too short to fully test all aspects of the approach, specifically in reaching beyond the involved managers to front-line staff and patients.

**Electronic supplementary material:**

The online version of this article (10.1186/s12913-018-3129-3) contains supplementary material, which is available to authorized users.

## Background

### Organizational improvement approaches in health and social care

Although healthcare quality is continuously improving worldwide, service providers are struggling to meet citizens’ rising standards and to provide guideline-recommended care to patients, all while simultaneously controlling costs [1, 2]. Continuous improvement (CI), defined as an organization-wide process of focused and sustained incremental innovation [3], in healthcare settings has led to varying results [4–6]. Challenges to CI in health and social care arise from issues of organizational size, complexity, context, and the loosely coupled systems and actors found at multiple levels [7, 8]. Calls have been made for both increased understanding of the interactions and inter-connections between organizational layers during change efforts [9] and for systems thinking [10].

Building the capability needed for CI in health and social care organizations continues to be difficult (e.g., barriers to fully embracing Lean as a system-wide organizational approach (e.g. [11–13]) or to use of the PDSA-loop to its full potential [14]). The need to involve senior management, work across functional divides, pursue value creation, and nurture a long-term view of CI has been emphasized [15]. Strategies to develop, integrate, and sustain CI approaches within and between organizational levels, however, remains under-researched. There is also limited research on how to achieve organization-wide strategies to aid the development of CI capability (e.g. [14, 16–20]). To fundamentally change organizational behaviors, basic underlying mental models must be addressed. Action-learning has been proposed as a way to address quality and safety challenges in healthcare and to foster double and triple-loop learning [21, 22]. Double-loop learning reframes or alters basic assumptions and values, leading to deeper and more sustainable change. In contrast, single-loop learning changes some actions or strategies, but not their foundations [23, 24] and triple-loop learning focuses on the structures and strategies used for learning (i.e., learning how to learn) [25, 26]. In action-learning, real-world problems are solved through concrete steps, while concurrently gaining deep learning in the process [27]. Action-learning is commonly described as including: a defined problem, an action-learning team, a process of emphasizing thoughtful reflection and listening, taking action, a commitment to learning, and a learning coach [28].

To differentiate our approach and to emphasize the learning process required to build CI capability, we refer to the development of *Continuous Organizational Improvement and Learning* (COIL) capability. In this article, developing capability refers to progress in COIL action strategies, knowledge, and competence possessed by key actors that decide on, affect, and support organizational development.

The purpose of our study was to develop and pilot-test a flexible, multilevel, multi-strategy approach for COIL capability building within a healthcare region in Sweden and to identify what was necessary to achieve changes in key actors’ approaches to support and work with COIL. We aimed to achieve changes in participants’ action strategies, knowledge, and competence, anticipating the need for double-loop learning. An action-research and action learning approach was used as we also wanted to identify aspects of the developed approach that had specific effects on participants. Further, we wanted to observe if potential new capabilities were used in development attempts involving other actors (e.g., staff and patients). Our intention was to improve strategies for supporting development of COIL in the case organizations. Even so, this was a first attempt and not a full evaluation of the developed approach. A full evaluation would require an alternative study design, including quantitative data to assess impact on service delivery.

### Characteristics of the COIL capability building approach

The first step in our development process was to decide on which attributes of our approach could be adapted to the organizations involved. We needed the approach to be flexible, yet consistently guide the building of COIL capability. The approach we developed was named “Sustainable Improvement and Development through Strategic and Systematic Approaches” (SIDSSA).

We assumed that a common strategy and basic level of competence were needed to effectively channel the inherent energy, development, and learning capability of an organization into action. Furthermore, we assumed that shared mental models held by actors on different levels would support COIL if the models were not overly rigid or detailed. Shared models, structures, procedures, and instruments were assumed to aid faster, more coherent and sustainable development processes within and between organizational levels. If team members’ capability in constructing and sharing mental models was enhanced, their team work and performance could be improved (e.g. [29, 30] Using shared approaches to handle multiple requests for change was assumed to provide synergistic effects.

The SIDSSA approach draws on three key components: 1) concurrent development on multiple organizational levels; 2) a pedagogical tool in the form of a development loop with five flexible phases, alongside example methods and instruments to support the phases of development; and 3) a learning approach that involves an action-learning loop for individuals and groups. We also pursued, on both individual and group levels, three main areas of competence and knowledge: a) improved systems views and systems knowledge, b) the use of systematic approaches to change and development, and c) increased knowledge and experience of learning and change processes within organizations, groups, and individuals. The multi-level systems approach (as opposed to a micro-system level focus) and the emphasis on promoting individual and organizational learning (double and triple-loop learning) differentiated our approach to others that have been used in health care quality improvement.

#### Strategies for multilevel development and multilevel action strategies

Ferlie and Shortell [31] outline the need for policy makers and practitioners to implement a comprehensive, multilevel approach to improve healthcare systems at the individual, group or team, overall organization, and larger system levels. Edwards Deming [32] discussed leadership and top management commitment as cornerstones for improvement, the requirement for profound system knowledge and understanding of variation, the theory of knowledge, and influence of psychology. Leaders’ support, including active sense-making aligned with organizational goals and visions, was highlighted as important for successful development [33]. Adequate time and resources, arenas for feedback and learning, and customer interaction were other identified key success factors for CI [33–36]. In addition, organizational practices, such as intensive vertical and lateral communication and high levels of delegation of decision rights, were recognized as needed, with employees rewarded for sharing and acquiring knowledge [37].

**Fig. 1:**
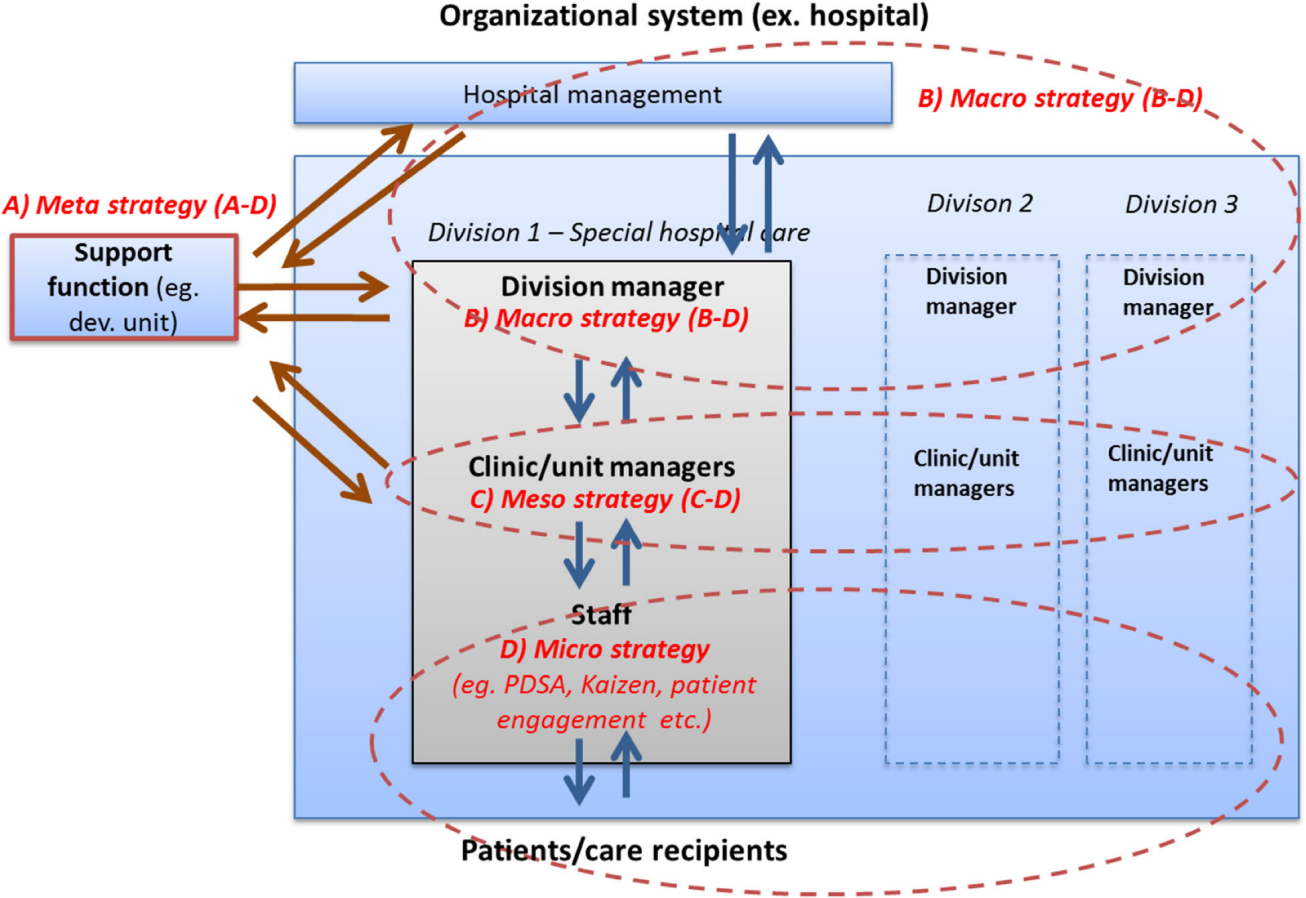
Multilevel strategies to build/support Continuous Organizational Improvement and Learning (COIL) capacity within an organization

In comparison, SIDSSA focuses on coherently strengthening the capability of the entire system, including a micro-strategy for staff-patient levels (indirectly aimed for in this study), a meso-strategy for unit managers, a macro-strategy for strategic managers, and a meta-strategy for support functions (Fig. 1). The developed approach acknowledges that care staff need strategies and procedures for improvement (e.g., small scale testing) that facilitate involvement of patients in problem solving, testing, and evaluation. Unit and strategic level managers need strategies to motivate, promote, monitor, evaluate, coordinate, and synchronize their unit’s efforts with larger organizational goals, strategies, and resources. Meanwhile, support functions need to understand the system, demonstrating both strategic and operative competency, and support functions and higher-level managers should be knowledgeable on all strategy levels to better facilitate the strengthening of COIL capability.

#### A flexible loop to visualize development processes on all levels

**Fig. 2:**
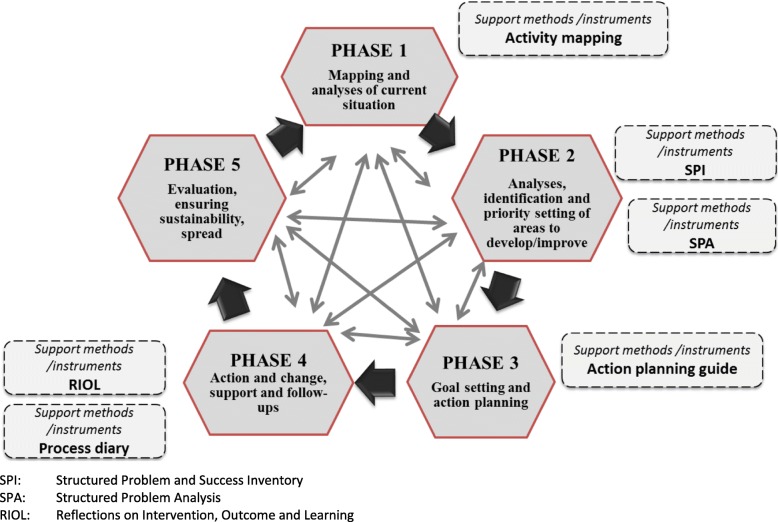
The development loop, including methods/instruments developed to support each phase, in the SIDSSA approach (Sustainable Improvement and Development through Strategic and Systematic Approaches)

Visualizing knowledge and processes is a way to enhance competence development, collaborative learning, and change (e.g. [38, 39]). We used a development loop (Fig. 2) with overlapping phases as a practical and pedagogical tool. The five phases in the loop are: 1) mapping and analysis of the current situation; 2) analysis, identification, and priority setting of the areas to develop; 3) goal setting and action planning, including plans for monitoring, feedback, and evaluation; 4) action and change, including support and follow-up; and 5) evaluation, ensuring sustainability, and spread. This loop shares many similarities with previous models related to change and development, for example the PDSA model [40], learning loops [23], and project management models [41], but puts greater emphasis on contextual understanding, problem analyses, and process planning. The development loop represents one way to describe and enhance a structured approach. For example, the role of active involvement in self-assessment prior to change, as a way of enhancing readiness for change [42], is highlighted (Phase 1 and 2). Further, a change facilitator may enter ongoing change processes to check if initiatives are based on appropriate information (Phase 1) and analysis (Phase 2) and if corresponding actions are in line with these (Phase 3).

Many methods and instruments have been created to support development (see [43] for examples). In cooperation with our participants, and to address their needs, new instruments and methods (described in forthcoming publications) were developed and tested. These included: 1) the Structured Problem and Success Inventory (SPI), which was designed to start analysis when describing problems or successes [44]; 2) the Structured Problem Analysis approach (SPA), which was designed to enhance development of shared mental models on an improvement issue and aid analyses, prioritization of issues, and action planning; and 3) the Reflections on Intervention, Outcome, and Learning (RIOL) instrument, which was designed to aid change facilitators when they are supporting development processes. Weekly process diaries, activity mapping, and structured activity planning were also used. Instruments and methods to aid each phase, including what was already in use by the organization, were flexibly chosen by the participants.

#### An action-learning loop connecting several levels

Batalden and Davidoff [45] view CI as dependent on five knowledge systems: 1) generalizable scientific evidence, 2) context awareness, 3) performance measurement, 4) plans for change, and 5) execution of planned changes. Monitoring and feedback of results are important facilitators of learning and change [34, 46]. However, achieving well-functioning monitoring and follow-up that aid COIL can be challenging and involves adherence to many dimensions [47]. Building COIL capability involves both single and double-loop learning on many levels (e.g., individual, group, and organization) [23, 24], implying changes in individuals as well as in unit and organizational culture. Learning organizations require multilevel engagement, capacity to learn, and several integrated, essential disciplines. These disciplines include: systems thinking, personal skills, mental models, a shared vision, and team learning [36, 48]. Coherence between structural and social aspects of organizations, processes, and competencies involved in COIL should also be considered [49]. An action-learning loop was the pedagogical model for the SIDSSA intervention [50]. It included active testing on improvement areas and supporting managers and staff in improvement activities, including various ways to enhance reflection and learning on both group and individual levels.

## Methods

A longitudinal, multiple case study design [51] utilizing qualitative data collection methods (i.e., interviews, process diaries, and archival data) was used to investigate the process and perceived effects of introducing SIDSSA within selected parts of a Swedish healthcare region. A case study approach has been previously recommended for longitudinal studies in natural settings where events, processes, and context cannot be fully controlled [51]. The three cases included in our intervention were: 1) a regional research and development (R&D) unit, 2) local support functions and division and unit managers involved in the care of the elderly (Municipality A), and 3) local support functions and division and unit managers involved in the care of adults and children with functional impairments (Municipality B). Convenience sampling of organizational cases was applied to strictly include cases motivated to develop their COIL capability.

Our study was part of the Future Welfare Services research project, which was initiated in October 2009 and focused on collaborative action research (AR) [52]. This report describes the overall procedure and results of the SIDSSA approach, while two others have focused on the action-research approach used by the researchers [53] and the detailed experiences and learning and change processes as perceived by members of the R&D unit [50]. Data was collected between December 2009 and March 2012 and involved interviews, weekly process diaries, and documents (see Table 1 for an overview of all data collected in the project).

**Table 1:**
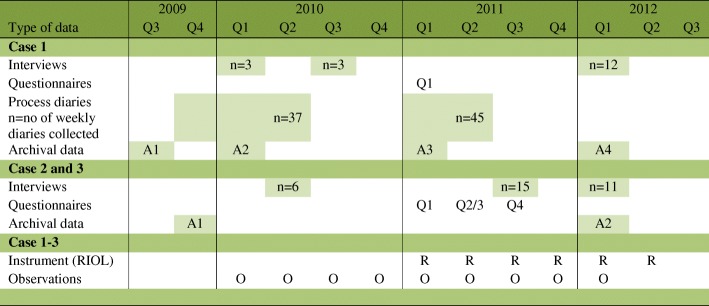
Overview of data collected in the Future Welfare Services Projecta, with the darkened cells indicating data used in the SIDSSA^b^ study

^a^Future Welfare Services projects (Grant no 2009–01729), funded by the research program on service and organizational innovations at Sweden’s innovation agency VINNOVA

^b^SIDSSA: Sustainable Improvement and Development through Strategic and Systematic Approaches

Case 1 refers to an R&D unit

Case 2 refers to “Municipality A” which provides care for the elderly

Case 3 refers to “Municipality B” which provides eldercare and care for adults and children with functional impairments

The R&D unit and Municipality A and B are blinded entities within one of the 21 healthcare regions in Sweden

As action-researchers, we mainly collaborated with members of the R&D unit. Responsibility for working with the municipalities was gradually transferred from the action-research team to the R&D unit. Table 2 provides an overview of the main SIDSSA interventions included in our study. Table 3 presents the SIDSSA approach’s underlying assumptions and theory of change.

**Table 2:** Overview of the main intervention activities included in the Future Welfare Services project^a^. The intervention activities are related to the five phases included in the SIDSSA^b^ development loop (see Fig. 2)

	2009	2010		2011		2012	
		Jan-June	July-Dec	Jan-June	July-Dec	Jan-June	July-Dec
	*Focus on:*	Phase 1	Phase 2–3	Phase 2–4	Phase 2–5	Phase 3–5	Phase 5
Case 1 R&D unit	• First contact and meetings with several levels • Planning and writing an application for funding	• Initiation • Meetings and workshops • Coaching via Skype and telephone • R&D mapping own context + Municipality A and B	• Two-day training retreat • Meetings and workshops • Coaching	• Meetings and workshops • Coaching	• Meetings and workshops • Coaching	• Meetings and workshops • One day workshop presenting experiences with other regional actors invited	• Meetings on sustainability • Reporting
	*Focus on:*		Phase 1	Phase 2–3	Phase 2–4	Phase 3–5	(….Phase 5)
Case 2 Municipality A		• First contact, initiation, and planning	• Seminars and workshops	• Two-day training retreat • Meetings and workshops • Coaching	• Meetings and workshops • Coaching	• One day workshop presenting experiences with other regional actors invited	• Reporting
	*Focus on:*		Phase 1	Phase 2–3	Phase 2–4	Phase 3–5	(….Phase 5)
Case 3 Municipality B		• First contact, initiation, and planning	• Seminars and workshops	• Two-day training retreat • Meetings and workshops • Coaching	• Meetings and workshops • Coaching	• One day workshop presenting experiences with other regional actors invited	• Reporting

^a^ Future Welfare Services projects (Grant no 2009–01729), funded by the research program on service and organizational innovations at Sweden’s innovation agency VINNOVA

^b^ SIDSSA: Sustainable Improvement and Development through Strategic and Systematic Approaches

Case 1 refers to an R&D unit

Case 2 refers to “Municipality A” which provides care for elderly adults

Case 3 refers to “Municipality B” which provides eldercare and care for adults and children with functional impairments

The R&D unit and Municipality A and B are blinded entities within one of the 21 healthcare regions in Sweden

**Table 3:** Description and underlying assumptions of the SIDSSA intervention

Interventions	Forms/Means	Rationale/Assumptions	Intermediate Outcomes Individual	Intermediate Outcomes Group/Unit	Long-term outcomes
R&D unit – regional development and change facilitators					
*Exemplify, test, and evaluate the usefulness of the SIDSSA development loop, the systems view and multi-level/multi strategy approach, and the regular use of reflective learning and action-learning loops* - On the R&D unit’s own improvement areas - On municipality cases A and B, involving participants from several hierarchical levels and phases in the development loop - Of specific work procedures and instruments on the R&D unit’s own improvement cases and municipality cases - On the participants’ own learning and change processes	*Researcher-led development:* - Workshops - Seminars - Group coaching - Individual coaching - Test on real cases - Visualization of process/structure - Joint collection of data/information - Joint analyses of learning process and reactions - Participation in adapting the approach to specific contexts and choices of arenas for testing	*- Action-learning loop for double-loop learning* *- Shared mental models* *- Systems view* *- Multiple levels, multiple strategies* *- Structured approach* *- Flexible and contextualized* *- Visualized development process* *- Increase complexity gradually*	*Improved knowledge* - Of the regional system of partner organizations, its levels, and functions - On change and learning processes *Improved competence, ability* - To act systematically when supporting COIL (map, identify, analyze, plan, execute, evaluate, sustain) -To analyze and reflect - To support others trying to change their work approach -To choose and use various procedures and instruments to aid change processes - Insights, attitudes, and behavioral changes in line with the new approach *Behavioral strategies, work approach* - Changed, more systematic, and strategic approach to support COIL - Clarified task, mission, and map of target organization	*Group strategies, work approach* - Common, systematic work approach to support and facilitate COIL *Shared knowledge* - Common team mental model of what it takes to change behavior and potential approaches to do so in their function	*Capability* - Improved capability as a unit to facilitate and support the development of COIL in the regional partner organization
Municipality A and B participants					
*Exemplify, test, and evaluate the usefulness of SIDSSA development loop, the systems view and multi-level/multi strategy approach, and the regular use of reflective learning and action-learning loops* - For unit managers with the participating unit’s own choices of improvement areas/projects - On participants’ own learning and change processes - For local support functions to test when supporting the participating units in their process - For division managers to support unit managers in their processes and to reflect on the usefulness of the learning for the whole division	*Researcher-led development:* - Workshops - Meetings - Follow-ups - Initial group coaching *Development led by the* R&D unit: - Meetings - Group coaching - Individual coaching - Follow-ups *Local test and development:* - Local development work and support within organization	*- Action-learning loop for double-loop learning* *- Shared mental models* *- Systems view* *- Multiple levels, multiple strategies* *- Structured approach* *- Flexible and contextualized* *- Visualized development process* *- Increase complexity gradually*	Improved knowledge - Of the organizational system and on-going development initiatives (national, regional, local levels) - On change and learning processes Improved competence, ability - To work systematically to support COIL in division and unit (map, identify, analyze, plan, execute, evaluate, sustain) - To analyze and reflect - To support others’ change and learning processes - To choose and use various procedures and instruments to aid change processes - Insights, attitudes, and behavioral changes in line with the new approach Behavioral strategies, work approach - Changed, more systematic, and strategic approach to support COIL	*Division strategy/work approach* - Development towards a common, systematic work approach to facilitate COIL in the participating units *Shared knowledge* - Common team mental model of what it takes to change behavior and potential approaches to do so in their function and in the division	*Capability* - Improved capability as a division manager; local support function and unit manager to facilitate and support the development of COIL in their organization

### Empirical setting

Health care systems in the Scandinavian countries have traditionally been decentralized. In Sweden, decisions on healthcare and in-home care are delegated to 21 county councils/regions and 290 municipalities, respectively. All are politically governed and independent. Our study took place in one of Sweden’s 21 healthcare regions. The region has 263,000 inhabitants and consists of a county council, responsible for healthcare, and nine municipalities, responsible for social services and in-home care.

A two-step convenience sample was used to select cases. First, we needed an intermediary function that had a mission to support development. The locally-based R&D units in Sweden provided such an opportunity [54, 55]. Therefore, we approached one R&D unit in a medium-sized region that had previously asked a member of our research team for input on how to develop their support strategies. After accepting our invitation, the R&D unit was asked to select two municipalities based on their experience and judgement of the municipalities’ readiness and motivation to try a new approach. Variation in the type of services provided was viewed as an opportunity to test the approach in different fields. The R&D unit served teams charged with elderly care and care of people with functional impairments. We chose one case from each area. The head of social services and a division manager in the two municipalities were then approached by the researchers. All agreed to participate.

#### The R&D unit – Regional support function

The R&D unit (Case 1) was formed in 2008 to serve the health and social care organizations in the region. Such locally-based R&D units are geographically spread throughout Sweden [54, 55]. The R&D unit’s mission was to aid competence and organizational development in the regional partner organizations and to conduct research in the areas of elderly care and care of adults and children with functional impairment. The R&D unit’s mission and operational plans were set by a regional committee, with representatives from ten partner organizations. Between 2009 and 2013, the R&D unit’s staff multiplied from three to 13, introducing new competences into the group. Five persons were specifically employed as development coaches in on-going national initiatives. During the scale-up of the SIDSSA intervention, the volume of the R&D unit’s assignments increased and the character of assignments changed. The changes in assignments arose, in part, due to more active selection of assignment in line with discussions related to the need for a new approach to the unit’s work.

#### Municipality a and B – Local support functions, division and unit managers within the sectors of care for the elderly and people with functional impairments

Municipality A (Case 2) had 32,428 habitants and 3025 employees. The budget for this sector, responsible for elderly care and care of people with functional impairment, was approximately 544 million SEK (€ 50,7 million) in 2010. Municipality A had two divisions: one that was responsible for care of elderly adults and the second focused on care of adults and children with functional impairments. The elderly care division covered ten special housing units and two larger units for older adults, who due to illness, required higher levels of care. A salutogenic perspective [56] was promoted by the division for some years and the R&D unit viewed the division as being ready to adopt new approaches and participate as a pilot case.

The division manager was asked to suggest a selection of lower-level managers (out of 24) that he/she thought would be interested in participating with the SIDSSA intervention. Five unit managers were asked to participate in our study and all accepted. After 5 months, one of these original managers was given responsibility for a large project and left our study. As a result, four managers were left to continue participate from 2010 to 2012. In addition, a more recently employed staff member, who held a support function, joined the project in 2011 and took on responsibility for supporting the SIDSSA intervention.

In 2010, Municipality B had 51,644 habitants and 3789 employees. This sector, responsible for care of elderly adults and care of both adults and children with functional impairments, had an annual budget of approximately 760 million SEK (€ 70,8 million). Health and social care in Municipality B (Case 3) had four divisions, mixing elderly care and care of adults and children with functional impairments. One division manager from this municipality participated in our study, together with five (out of 11) unit managers and two staff members that provided support functions. In 2007, Municipality B began promotion of a salutogenic perspective and implemented the International Classification of Functioning, Disability, and Health (ICF). ICF is a conceptual model and classification developed by the World Health Organization. ICF builds on a holistic approach, with interaction between bio-psycho-social model components [57, 58]. In early 2009, the R&D unit was assigned to assist with the ICF implementation. However, the interventions employed (seminars and educational sessions) had had limited impact by the time our study was initiated. Municipality B was perceived as ready for testing a new approach, with all of the contacted strategic managers willing to participate. In 2011, one of the support function participants retired.

In total, 12 persons, all women, from the two municipalities participated for the entire study period. An overview of the participants is given in Table 4.

**Table 4:** Overview of participants in Case 2 and 3 who were involved for the entire SIDSSA^a^ study period (December 2009 to March 2012)

Function	Years in this function	Unit	Number of staff supervised	No of care recipients
Case 2 - Care of elderly adults				
Division manager	2 years	Strategic management	24 unit managers	
Development support	1 year	Strategic level support unit	0	0
Unit manager	9 years	Three short term housing units	43	38
Unit manager	12 years	Special housing unit (dementia care)	22	24
Unit manager	> 10 years	Four special housing units	58	56
Case 3 - Care of adults and children with functional impairments				
Division manager	9 years	Strategic management	11 units	
Development support	8 years	Strategic level support unit	1340 staff	
Unit manager	11 years	Three housing units for people with autism	25 staff	17
Unit manager	20 years	Day-time occupation	70 staff	236
Unit manager	1,5 years	Three short term housing units (children)	30 staff	44
Unit manager	4 years	Home assistance and service	34 staff	8

^a^SIDSSA: Sustainable Improvement and Development through Strategic and Systematic Approaches

Case 2 refers to “Municipality A” which provides care for the elderly

Case 3 refers to “Municipality B” which provides eldercare and care for adults and children with functional impairments

Municipality A and B are blinded entities within one of the 21 healthcare regions in Sweden

### Data collection

#### Interviews

Data collection included a total of 48 semi-structured interviews. In Case 1, interviews were conducted three times with R&D unit participants, specifically in March 2010 (*n* = 3), September 2010 (*n* = 3), and December 2011/January 2012 (*n* = 10, the increased number of interviews reflects an increase in employees due to the previously mentioned hiring initiative). The semi-structured interviews aimed to capture participants’ views and learning processes, while addressing five key themes: 1) general development needs related to the SIDSSA intervention; 2) R&D unit development needs and obstacles, facilitators, and prerequisites; 3) individual staff member development needs; 4) change and development processes, preparation, monitoring, facilitation, and the actions taken/needed; and 5) perceived effects. The final interview was longer and focused on participants’ experiences with SIDSSA along the five identified themes.

Three series of interviews (*n* = 32) were conducted with participants from the municipalities (Case 2 and 3), in May 2010 (*n* = 6), November 2011 (*n* = 15), and March 2012 (*n* = 11). The first interviews were conducted by members of the research team and focused on capturing the initial situation within the municipalities and involved divisions. These were held with higher level managers (*n* = 3) and key strategic actors (i.e., support functions, *n* = 3). The questions included in the interviews addressed: 1) on-going priorities and projects, 2) support functions’ and key actors’ perceived importance attached to improvement work, 3) needs and challenges in the areas of providing care for the elderly and care of adults and children with functional impairments, and 4) perceptions and expectations associated with a new approach to improvement and development. The interviews held in November 2011 were conducted by R&D unit members. Transcripts from these interviews were used to validate findings and provide additional details for our analysis. Members of the research team conducted the final interviews with all participants still in office (*n* = 11). The final more detailed interviews covered: 1) participants’ function and role; 2) improvements conducted; 3) work approaches to development over time (previous/current/changes); 4) views on participants’ own role in development (previous/current/changes); 5) support (previous/current/changes); 6) SIDSSA processes, models, methods, and tools; 7) SIDSSA effects on development, the support structure, communication, cooperation with other staff members, work procedures, and care recipients; 8) expectations for the future and for different actors; 9) organizational learning; and 10) spread. All interviews were recorded and transcribed verbatim. Interview questions are provided as an Additional file 1.

#### Process diaries and documents

The R&D unit participants also wrote weekly process diaries during three periods (initial round of activities, testing period, and establishment of new approach). The diaries complemented our interviews. They contained details of the participants’ learning process and reflections on the changes tested, on-going change activities, and organizational learning processes (see also [52]), including obstacles and facilitators.

Action plans for the chosen areas of improvement (required in Phase 3 of the loop model), annual plans, annual reports, and other documents deemed relevant to capturing the process (e.g., web pages, PowerPoints, and filled in RIOL instruments) were obtained from all three cases. Approximately 30 documents were analyzed, mainly to provide contextual data for the participating organizations and to verify statements of time and content in plans, actions, and events as described in the semi-structured interviews and process diaries.

## Data analysis

The qualitative data were analyzed by three researchers (MEN, EH, JH) using conventional content analysis [59]. First, all interview transcripts, diaries, and documents were read through to get a sense of the data set. Then, each performed their analysis on a case by case, using all data sources and focusing on categorizing and describing: 1) the initial situation, needs, challenges, and expectations; 2) perceptions, experiences, and reactions during the development and learning process; and 3) perceived learning, effects, and outcomes. Throughout the analysis, consideration was given to the respondents’ role (regional and local support function, division and unit managers) to identify potential similarities or differences. When possible, several data sources (e.g. interviews, workshop posters, and action plans) were used to cross-check descriptions of activities, learning, and changes. In the end, the interview transcripts remained the main data source.

We strived for repeated measures (e.g., data repeated in both interviews and diaries) to capture the learning process and perceived changes. This was an invaluable part of the action-research approach and was used to guide adjustments in the SIDSSA intervention and adaptations in the coaching provided, improving fit during evolving contexts. Two researchers (MEN, EH) were actively involved in the intervention process. To avoid bias, other researchers were asked to double-check both basic data sources and analyses (JH) and to critically assess results and conclusions (RG, MAB, DT).

## Results

### The R&D unit – A regional support function

#### Initial needs and challenges

In the initial interviews and first series of diaries, R&D unit members reported a need for a work approach that better suited their mission to facilitate CI in partner organizations. They viewed their current work situation as fragmented and stated a desire to have improved methods to support partner organizations in building their capability for managing change. They also wanted to increase their own knowledge and competence in change processes. Finally, they looked-for increased knowledge concerning organizational systems, structure, and leadership. Developing these competencies was seen as a means to be able to better support and involve partner organizations in development and change activities, especially middle managers. An initial need to change the R&D unit’s role in the regional system was described. The R&D unit was working to become more collaborative and pro-active, promoting partnerships in regional as well as national initiatives. In summary, all participants urged improved strategies for planning during this period.

#### Change and development process

The R&D unit’s staff members’ development process when testing SIDSSA had two parallel tracks: one internally focused on the unit’s development issues and the second externally focused when. This dual track process was described as challenging, but also as one of the most valued assets of the intervention. Training under supervision of action researchers, in real settings, was mentioned as important for successful implementation, as was coordination between ongoing assignments and development activities. Yet, participants reported dilemmas when prioritizing between current commitments and the development process. Implementation was described as initially confusing and frustrating as participants struggled to understand the underlying assumptions of SIDSSA. An initial knowledge building process was therefore important, as was the openness of the R&D unit manager and municipal unit managers and support from and dialogue with the research team. Model illustrations, methods, and tools were also appreciated by the respondents. The stepwise nature of SIDSSA, and participants’ involvement during development of SIDSSA, was perceived as positive for the unit’s learning and change process. Specifically, participants emphasized the unifying effects of sharing the development journey and striving for common objectives and knowledge. Diary data provided individual details of the learning process, described by some as a “rocky journey”. This has previously been described in more detail by Höög [50].

*“You became very aware that it is so much easier to tell others to do certain things than to do it yourself. So when we had contact with others, this was when you started to think – what are we doing? Then it was easier to understand why they did not do things.”*

*“It has taken a lot of time and priority, but it has been positive - a huge potential. When we grew [in staff size] it was important to acquire a common way to work with our partner organizations.”*

#### Output – Capability building and new approaches

The R&D unit participants identified outcomes of the development process, which included a better understanding of systems and processes, more consistent and mission-aligned facilitation of partner organizations, and ongoing learning as strategic actors embraced SIDSSA. The process was seen as a unifying factor for the R&D unit, resulting in greater collaboration between staff, increased knowledge of assignments, and improved competencies. Participants also described better conditions for strategic and systematic thinking that improved their ability to handle task variation and new services requests. This resulted from clarified work approaches and improved support competencies. Data from documents, web-sites, and a PowerPoint presentation supported the respondents’ description of the new approach to work.

*“I think it has been smart and necessary. So much happened, new persons, changed assignments – it could have been anything, but since we worked systematically with this approach [SIDSSA] we could lead things differently and the R&D unit is really strong today.”*

### The elderly care division of municipality a – Local support functions and division and unit managers

#### Initial needs and challenges

Participants in Municipality A described several challenges and unmet needs that required a new COIL approach. Among the needs identified were: a holistic and sustainable strategic approach to learning and improvement, better follow-up, and ways to address staff members’ fatigue and obtain a motivated and engaged staff. Ability to fulfill their mission in providing care to the elderly, getting everyone to participate, implementing and holding on to changes, and enabling unit managers to work strategically with improvements, were also mentioned. Challenges described included: fragmented views on care for the elderly, vague goals, lack of leadership, ambiguities in mission for care providers and their managers, diversity in management competence, improper work conditions for managers to work strategically, and an absence of a forum for strategic planning. The participants expressed specific expectations going into the SIDSSA project, including gaining competency, particularly in being able to actively adapt to changes in mission, achieve focus on important issues, develop better leader role models, create a clear purpose and goals and useful measures of quality and gains during follow-up, and generate opportunities to discuss improvements and strengthen organizational values.

All participants saw the salutogenic approach as important. The new division manager expressed trust in staff members, describing them as willing and able to take on larger responsibilities. The new division manager also expressed a strong belief that a more systematic approach to improvement in the units’ daily work was possible. Existing facilitators mentioned included local support functions, inter-professional groups, and awareness of identified gaps.

#### Change and development process

Changes focused on health promotion in two housing units. The first unit was transitioning to a person-centered approach in short-term care and the second was working with a technical development project. Initially, some managers approached the implementation of SIDSSA with hesitation as the municipality recently had launched a separate model (called LOTS) for strategic management. This caused initial confusion as to which approach to apply (SIDSSA or LOTS). The issue was gradually solved in discussion with the R&D unit staff, identifying where LOTS fitted into the SIDSSA development loop. Almost all of the Municipality A participants mentioned staff member attitudes and values as influencing the change process. Referenced facilitators included group development, the units’ will to improve, focusing on “how to” (e.g., during Phase 1, there were many activities and seminars on measurement and how to combine measurement with a salutogenic approach), and the reflectiveness of staff.

According to the division manager, the speed of the units’ development was increased through the R&D unit’s support. Learning and spread was thought to be ensured by having all unit managers (> 20) participating in the initial SIDSSA phase. As a smaller team continued with the project, development in the larger management group slowed down. The recently hired support function staff member described herself as initially tentative, but after listening to the unit managers’ talk about SIDSSA, the holistic approach became more obvious to her.

According to the managers of the short-term unit, development of a person-centered approach initially met resistance among staff. This was especially true when routines and entrenched concepts (e.g., within a hospital setting, patients typically wear identification bracelets) were addressed. After a year, with educational seminars and process coaches addressing assumptions and attitudes, the situation gradually changed. The unit developed a collective view that reflected a more person-centered approach.

Managers of the special housing units focused a salutogenic approach; for example, by paying greater attention to clients’ needs and mapping their views of a meaningful day. The unit initiated a quality group that met regularly and included both care recipients and staff members from each ward. The managers supported each other and highlighted the R&D unit’s assistance. Vague project formulation was an initial obstacle that the units’ described overcoming.

*“We have a network group of volunteers 80 to 85 years old that are great. […] Staff that were responsible for entertainment, bingo, and gymnastics saw many barriers, but I told them to send out an inquiry; we cannot be certain that our elderly residents want to listen to accordion music […] Our goal is to become a health promoting eldercare unit. How did we work? We work with it [SIDSSA] all the time, focusing on it at staff meetings, away on educational sessions, and study visits. The driving force has been the quality group.”*

#### Output –capability building and new work approaches

According to the division manager, the SIDSSA approach provided a structured way of working, enabled identification of weaknesses in the eldercare organization, and supported the planning of new initiatives. By asking questions, more ideas from unit managers were obtained. Project goals were met, with the R&D unit contributing to a better understanding of how to support development and improve exchange between levels. The importance of planning, anchoring, and committing managers at different levels during the onset of change, and clarifying expectations to finish and follow-up on actions, were described as key. The importance of unit managers making development “their own” and engaging staff was emphasized. The support staff member described SIDSSA as her main approach for structuring work. One lesson learned was the need to clarify division of responsibilities, with unit managers as owners and leaders of development projects and support functions as supporters.

The pace in which unit managers adopted SIDSSA differed. One manager viewed SIDSSA as corresponding to previous ways of working and initiated a staff council to discuss and act on quality issues. The manager identified the importance of recognizing the need for development and devoting time to reflection and discussion before taking action. When defining action plans, she emphasized the importance of touching base and checking resources with higher management and political stakeholders before introducing plans step-wise to staff, and using the quality council for follow-up. The R&D unit’s support during Phase 1 was reported as strengthening motivation and aiding prioritization.

Other unit managers were more hesitant in adopting SIDSSA. Many used simplified or adapted models and instruments. Feedback from the research team and R&D unit staff was appreciated, but the local support function provided the most assistance. One manager expressed that SIDSSA increased her realization of the importance, as a manager, of initially expressing belief in development efforts, marketing ideas, and, when staff found their roles, stepping back and leaving room for staff to act. Managers at the housing units worked with mapping and the SPI and SPA instruments. Being repeatedly reminded that development is an on-going process and encouraging staff to keep the entire development loop in mind were viewed as aids to their work. Managers reported working in a more structured manner with development and remembering not to rush into action. One manager saw improvements in how they scanned the environment for new ideas and knowledge. Being able to share experiences was described as very positive for the group.

*“When I pose questions to the unit managers on what we should do and how to do it I get lots of ideas, other than saying this is what we shall do – an important lesson*”

*“Initially we imagined that we had not had much help from the SIDSSA project – of course we had, but we did not realize. In the beginning, we said that we can do this on our own. We do it our way, but it [SIDSSA] has fallen into place more and more.”*

*“The main lesson concerns the basic loop model, looking at different phases, where am I now and why am I there? Analyzing one’s own process, and not just results, is the biggest lesson learned.”*

#### Outcomes – Effects on units, staff, and elder adults

Unit managers provided several examples of the SIDSSA project’s effects on their unit, staff, and care recipients. At the short-term unit, changes in staff members’ language, attitudes, and routines (e.g., identification bracelets were no longer used on care recipients – who were now called customers) were reported. General facilities were improved (e.g., new bed linens and blankets were purchased) and welcome brochures for customers and their relatives were produced. Reactions from care recipients and relatives were positive, with comments made about the new warm, welcoming, and friendly environment.

At the special housing units, managers saw improvements in the conditions provided for the elder adults in their care, with a larger variety of activities offered. Instead of staff deciding on services and activities, staff asked for and tailored offers based on customers’ input. Many suggestions made by the care recipients were fulfilled (e.g., an art course and fashion shows were held). Staff, in general, began to understand the importance of working from a person-centered perspective. Improved group dynamics and cooperation in the quality group were also reported.

*“In one ward, with seven staff, each staff member has their own area of responsibility, areas they are interested in. One person will once a week play the piano for the old people; another is a painter and will paint and do ceramics with them. One leads singing, one provides massage therapy, and one is responsible for the library and will once a week borrow books and read to the residents. We have come a long way.”*

### Municipality B’s care of adults and children with functional impairments division – Local support functions and division and unit managers

#### Initial needs and challenges in the local context

Participants from Municipality B reported the need for development of long-term strategy, challenges associated with a new generation of care recipients with different abilities and demands, the need to address competition from private caregivers, unit managers’ desire to promote better work conditions for staff, requirements for improved communication, and a desire for greater cooperation and for meeting care recipients’ needs. Recruiting educated staff, down-sizing and job cut-backs, turn-over of managers, and difficulty communicating what to do once goals were reached, were reported as challenges. Additional challenges included: unit managers’ struggle to work on improvement, lack of follow-up, the large number of incidents in quality reports, the need to keep up-to-date with all approaches/tools available for each care recipient, requirements for knowledge transfer between staff, and the need to achieve common values and understanding of the core mission.

In addition, Municipality B participants described multiple expectations around what the SIDSSA approach could achieve. Participants looked for opportunities to encourage staff members’ active contribution to organizational development; establishment of a common project model and long-term strategies; follow-up, support structures, and strategies for sustaining good work; and development of an understanding of the relationships between research, knowledge, and success. Implementation of yearly quality measurements; improved communication and teamwork; development of joint goals; focus on care recipients; provision of safe and high quality care; advancement of the unit’s reputation; and a clear, shared value base; were additional expectations. Finally, ICF was expected to be a tool for systematic work on several levels: as both a model for care and a way to improve communication. Participants reported increased structure in implementing ICF, developing staff members’ ICF-thinking, and understanding each individual under their care.

Participants also described support structures in their units’ development, including: laws, guidelines and routines, quality policies, tools and follow-up, quality conferences, Institute for Healthcare Improvement (IHI) breakthrough methodology specialists, special support units, SIDSSA project support, social pedagogues, strategic documents covering measurement, and individual and group coaching. A final support structure was the inspiration provided by individuals involved in the change processes.

#### Change and development process

The areas chosen for development varied among the units in Municipality B, but documentation and implementation of ICF was often included. The division manager focused on ICF organization and implementation and on building a network organization. The daycare unit experienced a continuous flow of people. As a result, the manager chose to improve strategic planning, the process of receiving new care recipients, and tailoring service delivery. The short-term housing unit for children and young adults focused on improving ICF documentation. The manager and staff responsible for personal assistance and home-based services for care recipients focused on documentation and the process of developing, adhering to, and updating individual service plans. The newly employed manager for the special housing unit for individuals with autism concentrated on improving service quality, staff values, staff interaction, and the pedagogy employed. The support function staff member used SIDSSA to organize and plan for an up-coming educational program.

Coaching was performed by R&D unit members familiar with ICF. The change process was positively described by several participants, especially by the division manager and the support staff member. Both worked actively to spread information on SIDSSA and its progress. Decisions that created more time were described as facilitating development processes. One manager expressed that it was easy to work with SIDSSA once she took time to understand the model.

Perceived barriers for development included a shortage of time and choosing extensive areas for improvement. One manager saw barriers in being new to her position, having too much work, not knowing enough about the SIDSSA project, and not investing enough time for engagement. Another manager described having to work with different strategies in different units, with change processes causing frustration and a drain of energy.

*“In this ICF project, I had the privilege and opportunity to discuss and receive coaching. This has been very beneficial and a great difference from before.”*

*“I prepared a slide with the different phases of the loop and showed my colleagues and boss. It was time for our unit to reflect, because we run too fast. I wanted us to try this approach.”*

#### Output –capability building and new work approaches

The division manager in Municipality B recognized that SIDSSA provided a shared model, tools, and “*language for communication*” that were key to successful development, alongside systematic documentation. The experimental learning experience, i.e., the connection to and support from action-researchers, was also appreciated. She identified the coaching provided as a strong benefit and clear difference from other projects. During the study period, expectations for unit managers to initiate and support development were clarified. The close participation of staff in the change process, spread of the model, and the subsequent ability of participants to speak the same language were described by the support staff member as important. SIDSSA was further described as a valuable way to think and work. Changes were specifically noticed in the way unit managers communicated.

Unit managers described their experiences and lessons learned slightly differently. Although the unit managers described the model as useful, by study end, the SIDSSA had not spread to all unit staff members. The manager of the special housing unit for people with autism discussed the importance of the units’ group processes. SIDSSA provided support and a new way of thinking about leadership-style and group development phases. The approach spread as “*a culture of how to work in the units, both consciously and goal-directed*.” SIDSSA also highlighted a path forward in all phases and at each level, fitting closely with the way the unit manager wanted to work. The loop model was pronounced to be simple to use and applicable in many situations.

The benefit of SIDSSA from the daycare unit manager’s perspective is that the approach made her slow down, reflect, and consider alternatives rather than rushing into “quick fixes.” Mapping, analyzing, and considering the complete situation was the main lesson learned and new strategy employed. The manager of the short-term housing unit for children and young adults emphasized SIDSSA as an inherent way of thinking, but she often skipped several steps and had to learn to reverse, start again, and be less hurried. She described further lessons, including recognizing that many things can influence a situation, the necessity of taking time to think before acting and implementing change, not overly stressing change, and making sure your focus is on the right things.

The manager responsible for personal assistance and home-based services for care recipients viewed SIDSSA as a highly personal experience and an eye-opener. The manager had recently been promoted from a staff position and had attended leadership education. SIDSSA helped her document her thoughts and supported her transition to greater responsibility.

*“An awakening experience to view one’s work differently at the unit, understand more how to reach out and promote changes, or talk when something needs to be done. It is not easy, but when you have a systematic way of working during change processes, it has been very precious.”*

*“SIDSSA is in many ways a way of thinking that I have with me, but I often skip a few steps because everything happens so fast. So I had to reverse one or a few steps. […] By participating in the project, I recognized that I do this and I’ve been forced to think and try to reverse.”*

#### Outcomes – Effects on units and staff that provide services

The greatest number of changes occurred in the special housing unit for people with autism, which focused on developing values, pedagogy, and service quality. Conversely, the units that focused largely on documentation described themselves as being in earlier development phases, with fewer examples of changes they had successfully made by the end of the study period. The manager of the special housing unit for people with autism, however, described improvements for care recipients, specifically the materials and pedagogy used, and staff members’ analysis and management of incidents. For the daycare unit, mapping clarified the need to increase efforts to find work opportunities for a target group of care recipients (a coordinated function to identify work had recently been introduced). The manager of the short term housing unit for children and young adults was still in the process of improving documentation and expressed an anticipated outcome of care recipients receiving more coherent care. The manager of the unit providing personal assistance and home-based services for care recipients described improvement of care plans as an on-going project. After technical problems, all her wards now had computers – a basic resource.

*“There is so much material now for the care recipients. Before, staff worked towards the target group and knew that they should work with clarity and clarifying pedagogics. […]This was not visible and if I, as a manager, cannot see it – how will the care recipients? […] Just setting up a goal for a care recipient – and then talking about how we can reach this goal.”*

## Discussion

The purpose of our study was to develop and pilot-test a flexible, multilevel, multi-strategy approach for COIL capability building within a Swedish healthcare region and further, to identify what it would take to achieve changes in key actors’ approaches to support and work with COIL. Our study contributes to the call for research on developing CI capacity and the need to build system-wide approaches and strategies for increasing key actors’ competencies in organizational improvement and learning [14, 16–20]. New approaches are needed to address vital challenges in health and social care related to size, complexity, and context; working with loosely coupled systems and actors [7, 8]; and simultaneously pursuing quality, safety, and cost controls [1, 2]. Our study also contributes to the knowledge base surrounding how to support organizations in changing deep-rooted behaviors through action-learning and cultivation of double and triple-loop learning [21, 22]. The study’s pilot project led to important lessons, further described under the first four headings below, including specific aspects of change that need additional consideration by change agents seeking to build COIL capability in health and social care.

### Pre-requisites for initiating capability development – Motivation and readiness

As in all change processes, motivation is key. All three cases demonstrated an initial readiness for change and were included in our study based on this criterion. R&D unit members described the need to better fulfill their mission by changing their work approach and their role within the regional system. In the municipality cases, identified needs included better support of managers so they could focus on motivation of staff and facilitation of improvement activities in their units. As such, the observed development and learning outcomes were achieved by a group of initially motivated participants. In previous work, assessing readiness for change, before initiating change, has been described as an important first step [60]. In practice, instruments developed to measure organizational readiness can be time consuming and too general, overlooking intervention-specific and local context information that often is needed.

Mapping and discussion of challenges and needs in relation to current and anticipated situations provided motivation and insights into the action strategies used by participant groups and individuals. Mapping provided a sense of coherence and control for the municipality participants, as well as an understanding of the competencies needed. For example, we observed an initial lack of competence in micro-level strategies among most participants. This was viewed by the R&D unit members as a significant barrier. Being involved in self-assessment prior to change may enhance motivation and, hence, readiness for trying new approaches [42]. However, mapping cannot be overly time-consuming in relation to the onset of action. If time is limited, introducing a time-consuming mapping process will negatively affect the overall change processes. This may, in part, explain the mixed results found within the municipality cases.

### The use of a systems approach – Multi-level strategies and actors from multiple system levels

Our plan was that the R&D unit would focus on aiding managers and local support staff to work more systematically and strategically in development activities. In turn, the managers would aid their staff in developing new improvement approaches. At the start, participants were given flexibility to choose improvement areas and the pace of development. It was deemed necessary to internalize and make the overarching COIL approach their “own” model for improvement. These tactics were guided by reflections made by both the R&D unit staff members and the managers and involved changes in both roles and the organization’s culture in relation to improvement and learning. The first step was to achieve double-loop learning by key individuals. Our results indicated that participants’ basic assumptions on how to work with change transformed and a shared mental model of the change process developed. In the R&D unit, a new way of working with facilitation also evolved. Shared mental models can be seen as steps towards change in organizational culture. Further guidance for unit managers and staff in moving forward to develop micro-strategies could be provided, in future work, through educational sessions based on the PDSA model, the use of iterative cycles, the need for small-scale and prediction-based testing of change, the use of data over time, and the value and best practices for documentation [14].

Meta-strategies for the R&D unit and meso-strategies for unit managers were in focus during the intervention, while management approaches on the division level (macro-strategies) were still under development when our study ended. More time would be needed to ensure sound conditions for sustainability, especially in the municipalities. For example, by the end of the study period some unit managers had not involved their staff in the development process and full involvement of upper, strategic management levels had not been reached. In general, more interventions and time were needed to facilitate development strategies at higher levels within the health and social care systems. The R&D unit’s meta-strategies were found to include an action-research component, with unit members’ assignments designed, documented, and presented scientifically. Full realization of the action-research component was not achieved, partly due to time and resource limitations.

When the intervention ended, the SIDSSA approach had not reached all involved levels of the municipalities, creating a threat to sustainability. To ensure adaptation to contextual factors and participants’ needs, the participants chose the improvement areas for testing. The choices to focus on rather large development issues surfaced as a factor that reduced the speed of change. In Municipality B, involvement by the division manager decreased over time, additionally slowing the process. The competencies of many of the managers remained untested as they had not had an opportunity to apply their new insights and strategies with their staff members. This is an important step where additional coaching would have been beneficial. In retrospect, including managers who were new in their role, and participants on the way to retirement, was not optimal. Nevertheless, strategic management involvement, coaching, and working with managerial colleagues enhanced the observed change and learning processes.

In our study, two system levels of the region’s support structure were involved – the regional R&D unit and the municipalities’ own support functions. The project provided an arena for R&D unit members to introduce themselves. Before the project, many municipal managers were unaware of the R&D unit’s existence and services. The R&D unit reframed its mission and gained additional insights into its role in the regional system, learning valuable lessons about how their partner organizations functioned. These lessons, along with mapping of on-going initiatives at the national, regional, and local levels in the involved care areas, provided a comprehensive overview that represented an attempt to both gain and provide a holistic view of the organizational system. Both internal and external communication increased and improved in clarity. The R&D unit’s approach to new assignments or requests from partner organizations also changed, partly due to SIDSSA and partly due to national initiatives with additional service requests [4].

### Enhancing learning by visualization of development processes - the generic development loop

Both R&D unit and municipality participants found the structure of the SIDSSA development loop helpful. To use and visualize structures for development processes was rather new to this group, and was, in fact, the main learning outcome for the municipalities. The SIDSSA development loop, and the long, two-year learning process, helped participants obtain a mental model that aided interpretation and supported actions taken during development. In many cases, the participants’ mental models became a shared or team mental model, further enhancing change [29, 42]; buttressing creativity, learning, and innovative knowledge creation [61]; and facilitating communication and coordination [62].

The R&D unit’s development of systematic ways of working was represented by actions taken in line with the SIDSSA development loop, e.g., where documentation was improved in all cases. That said, documentation remains a main challenge. The simplicity of the SIDSSA development loop was viewed as an explicit advantage, with the systematic approach of SIDSSA viewed as supporting successful development. For successful development, both simplicity and an understanding of complexity were needed.

### Action-learning approach and sustainability – Providing time for double-loop learning

Hands-on testing was important for the R&D unit participants’ development, but keeping pace with the learning process in the municipalities was challenging, specifically when using intermediate actors and when promoting several learning processes simultaneously. This supports the previous observation by researchers that “learning by doing” is a strong facilitator (e.g. [21, 22, 25–28, 63]). Ways to assist change in support functions should be described as a continuum, starting from a discrete task-focused activity (i.e., doing for others) and leading to a process of enabling individuals, teams, and the organization [64]. Furthermore, important insights into change and learning processes, the time and effort needed to change habits and work approaches (i.e., double-loop learning) of individuals and groups, and the different phases of change and how these phases might express themselves, were all indicated by the R&D unit as new process knowledge competencies they gained that were important in their change facilitating functions [65].

Despite shortcomings in our project’s reach and probable limits in sustainability, specifically in Municipality B, similar insights on change processes were achieved in both municipalities. However, these insights were not as elaborated as within the R&D unit, where the SIDSSA approach was initiated 6 month earlier. The R&D unit embraced the SIDSSA approach and, in addition to the two pilot cases, continues to use the approach in other development projects. Even so, much work remains to fully establish COIL capability in the region’s health and social care sectors.

### Methodological considerations

To measure learning processes and changes in assumptions, and the competence and capability of individuals and groups, is not easy. We focused on what was learned and if major assumptions of participants were altered, realizing that everyone started from different levels of competence and readiness for change. We used complementary data sources to provide input to our analyses, with efforts made to crosscheck sources when possible. However, to assess changes over time, it would have been beneficial if we had been able to include quantitative measures. The open approach we took, specifically in the local units, meant that participants tried out their developing capabilities on very different areas, in different contexts, and at different paces. This made it difficult to assess outcomes in terms of changes achieved at the units. Here, we relied on participants’ descriptions of their development work and what they and others described in interviews and documents.

When using a case study approach, generalization is an issue. Suggested solutions include the use of multiple cases and reliance on theoretical generalizations [51]. In our study, we used multiple cases that all tested the same general SIDSSA approach. Still, all three cases came from the same regional and national context, limiting the generalizability of our results. Further evaluation of the SIDSSA capability building approach in other contexts, and with different cases, is needed. Such studies would benefit from a longitudinal design, utilizing both qualitative and quantitative methods, to further assess interventions as well as intermediate and longitudinal outcomes.

## Conclusions

A common understanding and shared mental models of how to approach development initiatives and change within organizations can be keys aids to organizational learning and development [20, 36]. As exemplified throughout our study, an overview of the organizational system, its context and on-going change initiatives, and having a structured approach to handle change, provides a sense of managerial control, making it easier to initiate and support improvement and learning. A simple, generalizable model that can easily be understood and internalized by key organizational actors is also likely an important initial step, before more complex models for specific development and improvement can be implemented. In addition, the use of a systematic approach and capability building strategy involving several hierarchical levels was important for the learning process as complementary strategies are needed on several levels. We concluded that having a long-term perspective and ‘contract’ was needed to develop COIL capability and facilitate the development process. Based on the initial organizational situation and competence levels, further development of COIL capability may take more or less time. In our study, there were no coherent organizational strategies for how to support development in the region, in the municipality, or in the division. It was primarily the responsibility of unit managers to find ways to achieve change. We found that involving regional and local support functions together, including managers at different levels, and working actively with real cases chosen by participants, enhanced a systems view, the learning and development processes, and, potentially, the sustainability of change. These findings are relevant for managers and decision makers operating in various health and social care systems as they focus on increasing the capability for improvement and learning in their organizations and systems.

## Additional file

Additional file 1: Interview manuals. Interview manuals for three rounds of interviews with participants from the municipalities (Case 2–3) and the three rounds of interviews with members of the R&D unit. (DOCX 27 kb)

## Abbreviations

CI: Continuous improvement
COIL capability: Continuous Organizational Improvement and Learning capability
ICF: International Classification of Functioning, Disability and Health
R&D unit: Research and Development Unit
SIDSSA: Sustainable Improvement and Development through Strategic and Systematic Approaches
