# Supplementary material for: Exploring the potential of a multi-level approach to improve capability for continuous organizational improvement and learning in a Swedish healthcare region
Source: BMC Health Serv Res. 2018 May 24;18:376. doi: 10.1186/s12913-018-3129-3 (PMC5968489; doi:10.1186/s12913-018-3129-3)
Supplement: Supplementary file 1 — Interview manuals. Interview manuals for three rounds of interviews with participants from the municipalities (Case 2–3) and the three rounds of interviews with members of the R&D unit. (DOCX 27 kb) [file 12913_2018_3129_MOESM1_ESM.docx]

# Additional file 1 – Interview questions

## Abbreviations

FWS – The Future Welfare Services research project

SIDSSA - Sustainable Improvement and Development through Strategic and Systematic Approaches

## Interview 1 – Municipalities May 2010

Higher level managers and strategic actors were interviewed using a semi-structured interview manual with openness for the respondents to address themes/aspects in more detail depending on position and for the interviewer to follow up on the information provided. These were the questions we aimed to get information on:

1. Can you describe the priorities and overarching goals that you currently are working to achieve in your area in the municipality?
2. Can you describe the projects or initiatives that currently are or recently have been in focus in your area in the municipality?
3. Can you describe the strategies for working with development and improvement? Arenas?
4. What are your experiences of working with improvement? Good examples?
5. How do you view your role in relation to development?
6. What support is there for work with development and improvement?
7. What are the needs and challenges you currently face in your area in the municipality?
8. What are the difficulties you currently experience?
9. What are our expectations on a new approach to improvement and development?

## Interview 2 – Municipalities November 2011

The interviews held in November 2011 were conducted by R&D unit members (some researchers themselves) supervised by the research team. This was a learning opportunity and provided an example on how to get information on a development process and how to act as an action researcher. Transcripts from these interviews were used to validate findings and provide additional details for our analysis.

1. What in your opinion is it that will be developed by the FWS project?
2. What kind of development do you want to see for the unit?

- What do you see as the necessary conditions for such development?
- What barriers do you see for such development?
- What enablers do you see for such development?

1. What kind of development do you want to see in your role and function?

- What do you see as the necessary conditions for such development?
- What barriers do you see for such development?
- What enablers do you see for such development?

1. What kind of development do you want to see for the region?

- What do you see as the necessary conditions for such development?
- What barriers do you see for such development?
- What enablers do you see for such development?

1. What development do you want to see for Municipality Case A and Case B?

- What do you see as the necessary conditions for such development?
- What barriers do you see for such development?
- What enablers do you see for such development?

1. What role does the external research team play in the on-going development?
2. What measures/actions have been taken at the R&D unit to give room for the development (FWS/SIDSSA)?
3. Are there any more measures/actions needed?
4. What do you think you have learned so far (since the FWS-project started)?

## Interview 3 – Municipalities March 2012

***A. Function/role***

1. What type of area are you working within? What is our function?
2. How long have you worked as a unit manager/division manager/support function?
3. How many staff and residents/patients are you responsible for in your area/unit?

***B. Conditions/prerequisites and needs for development/improvement work***

1. Which development and improvement initiatives have you/and your staff worked with during the FWS-project period?
2. Where are you today in these development/improvement initiatives?
3. What is most important to develop within your unit or area today and in the future?
4. Which are the important conditions/prerequisites that affect development/improvement work in our unit/area?

***C. Approaches used for development/improvement work - Previous, Current, Effects***

1. How would you describe the approaches and strategies for development/improvement work you used within your unit/area before the FWS-project?
2. Have there been any changes in how you yourself and your unit/area work with development/improvement work compared to before you joined the FWT-project?
3. If yes – which ones (describe)?
4. If yes – what initiated/affected this change?

***D. Own role and views on development/improvement work and support – Previous, Current***

1. Can you describe which role you in your function have for the work on development/improvement in your area/unit? What is important for you to work with?
2. Have the FWS-project and the SIDSSA approach affected your views on development/improvement work? If yes – How?
3. What support do you perceive you have for working with development/improvement work? What support would you need/or need to further develop?
4. What do you think is important to be able to make co-worker/staff develop new work approaches?

***E. SIDSSA – Process, methods, tools, instruments***

1. Have you used any tools/instruments introduced during the FVT project period? If yes, which ones (describe)?
2. If yes – How have you used them?
3. If yes – How do you think they have worked out? Advantages? Disadvantages?
4. Do you intent do use any of them in the future? (which ones)

***READ: SIDSSA aimed to support a holistic or systems view, strategic and systematic approaches and contribute the increased knowledge on how to work with development, improvement and learning processes.***

1. Do you think that the work with FWS/SIDSSA during this period have contributed to these aims for you or your division/unit? If yes can you give some examples?
2. Have you seen any effects on your co-workers/staff after working with the SIDSSA approach in the development/improvement initiatives? If yes can you give some examples?
3. Have you seen any other effects on residents/patients our in the work performed at the unit after working with the SIDSSA approach in the development/improvement initiatives? If yes can you give some examples?
4. Have the work with the SIDSSA approach affected communication or collaboration in any way? If yes – how (describe)?
5. Is there anything that has made the work with the SIDSSA approach more difficult during the project period (hinders)?
6. Is there anything that has made the work with the SIDSSA approach easier during the project period (enabler)?

***READ: The FVT project and the SIDSSA approach had also the intention to contribute to the building of an organizational support structure – from regional level via division level to unit managers and eventually to staff.***

1. Do you think that the work with FWS/SIDSSA during this period have contributed to these intentions for you and your unit/division? If yes can you give some examples?
2. After participating in the FWS-project – What reflections and expectations do you have on the R&D unit’s role?
3. After participating in the FWS-project – What reflections and expectations do you have on the role of the division management?
4. After participating in the FWS-project – What reflections and expectations do you have on the unit manager role?
5. After participating in the FWS-project – What reflections and expectations do you have on researcher’s roles in improvement and development work?
6. Of the things that have been introduced by the FWS project/SIDSSA approach do you think you will continue to use in future development and improvement work?
7. What are the most important learnings that you and the division/units have had during the FWS project period and when you have tested the SIDSSA approach? How do you or your unit intend to proceed with these learnings?
8. Is there anything that you have planned to spread further? How?

***H. Other***

1. Is there anything else ou would like to adress in relation to what we have talked about in the interview?

## Interview 1-2 - R&D unit members - March and November 2010

1. What in your opinion is it that will be developed by the FWS project?
2. What kind of development do you want to see for the unit?

- What do you see as the necessary conditions for such development?
- What barriers do you see for such development?
- What enablers do you see for such development?

1. What kind of development do you want to see in your role and function?

- What do you see as the necessary conditions for such development?
- What barriers do you see for such development?
- What enablers do you see for such development?

1. What kind of development do you want to see for the region?

- What do you see as the necessary conditions for such development?
- What barriers do you see for such development?
- What enablers do you see for such development?

1. What development do you want to see for Municipality Case A and Case B?

- What do you see as the necessary conditions for such development?
- What barriers do you see for such development?
- What enablers do you see for such development?

1. What role does the external research team play in the on-going development?
2. What measures/actions have been taken at the R&D unit to give room for the development (FWS/SIDSSA)?
3. Are there any more measures/actions needed?
4. What do you think you have learned so far (since the FWS-project started)?

## Interview 3 – R&D unit – December 2011/January 2012

**A) The SIDSSA approach – Understand – Use – Value - Spread**

*We start with the overall* ***SIDSSA approach***

1) How would you describe the SIDSSA approach?

2) In what areas/situations do you think it can be used?

3) What possibilities and difficulties do you see with SIDSSA as an approach to development?

*We continue with the* ***SIDSSA development loop (and its phases)***

4) How would you describe the development loop?

5) In what areas/situations do you think it can be used?

6) What possibilities and difficulties do you see with the development loop and its use?

*We introduced some* ***methods/tools/instruments*** *related to the phases of SIDSSA development loop)*

7) Have you used any of the introduced methods/tools/instruments? Which ones?
8) How would you describe the introduced methods/tools/instruments? (the ones you have used)
9) What possibilities and difficulties do you see with the methods/tools/instruments?

10) What do you think about the spread of the methods/tools/instruments?

*SIDSSA’s* ***multi-level strategies*** *(micro, meso, makro, meta)*

11) How would you describe the multi-level strategies?

12) What do you think about the use and spread of the multi-level strategies?

*SIDSSA focuses on* ***development of three main areas of competence and knowledge****: a) improved systems views and systems knowledge, b) the use of systematic approaches to change and development, and c) increased knowledge and experience of learning and change processes within organizations, groups, and individuals.*

13) How would you describe these areas?

14) How has the work with FWS and the SIDSSA approach contributed to these three areas?

15) In what situations/circumstances has the R&D unit used these three areas?

16) What possibilities and difficulties do you see when working with these three areas?

*The R&D unit’s* ***internal development areas*** *(internal competence development, how to deal with new missions, improved information-communication)?*

17) How have you worked with the three areas?

18) How have you used the SIDSSA approach during your work with the development areas?

19) What possibilities and difficulties have you seen with the use of the approach for internal development?

20) Do you think the approach will be used for future internal development within the R&D unit? If yes – How?

**B) The development process during the FWS-project (learning/implementation)**

21) How has the development process affected the R&D unit as **a function**?

22) How has the development process affected the R&D unit as **a group**?
23) How has the development process affected you **personally**?

**C) Results/effects/changes due to FWS/SIDSSA**

24) What are the most important results/effects/changes of the FWS project for you as an **individual**?

25) What are the most important results/effects/changes of the FWS project for the R&D unit as a **group**?

26) What are the most important results/effects/changes of the FWS project for the R&D unit as a **function**?

27) What are the most important results/effects/changes of the FWS project for the **municipalities (cases)** – from higher levels to residents/patients?

28) What are the most important results/effects/changes of the FWS project for the **regional level**?

**D) The action research perspective**

29) How do you perceive the action researchers role in the development process during the FWS-project?

30) How do you perceive the action researchers role in relation to the effects of the FWS – project?

31) How do you perceive the Research and the Development in the R&D unit for the future? What is desired? What will probably happen? What do you fear?

**E) Questions for the newly employed – entering during an on-going development process**

32) In what way where you introduced to the FWS-project and the SIDSSA approach?

33) Did you get access to any written information on the FWS-project and the SIDSSA approach?

34) What do you need to be able to learn and use the SIDSSA approach?

**F) Other**

35) Do you have anything more you would like to add?
